# Incidence of Long-Term Esophageal Dilation With Various Treatment Approaches in the Older Head and Neck Cancer Population

**DOI:** 10.3389/fonc.2018.00466

**Published:** 2018-10-23

**Authors:** Garrett Green, Ellen Kim, Ruben Carmona, Hanjie Shen, James D. Murphy, Loren K. Mell

**Affiliations:** ^1^Department of Radiation Medicine and Applied Sciences, University of California, San Diego, La Jolla, CA, United States; ^2^Department of Radiation Oncology, Vanderbilt University, Nashville, TN, United States; ^3^Department of Radiation Oncology, Abramson Cancer Center, University of Pennsylvania, Philadelphia, PA, United States

**Keywords:** LAHNC, incidence of esophageal dilation, chemoradiotherapy, long-term esophageal dilation, treatment

## Abstract

**Purpose:** Treatments for locoregionally advanced head and neck cancer (LAHNC) negatively impact swallowing function, but the long-term incidence of severe toxicity requiring esophageal dilation is not well-documented in the population. The aim of this study was to compare the incidence of long-term esophageal dilation across varying treatments for LAHNC.

**Methods and Materials:** We identified 5,223 patients with LAHNC diagnosed from 2000 to 2009 in the SEER-Medicare database. We compared the incidence of esophageal dilation for surgery alone vs. surgery plus adjuvant radiotherapy (RT) and chemoradiotherapy (CRT) vs. definitive RT or CRT.

**Results:** The cumulative incidence of esophageal dilation for all sites at 10 years, according to treatment group were as follows: CRT, 14% (95% confidence interval (CI), 12–17%); definitive RT, 13% (95% CI, 10–16%); surgery alone, 5% (95% CI, 3–7%); surgery and CRT, 15% (95% CI, 11–19%); surgery and adjuvant RT: 10% (95% CI, 8–13%). There was no significant difference in the incidence of esophageal dilation between surgery plus adjuvant RT/CRT or definitive RT/CRT (*p* = 0.37), but the incidence was significantly increased in both groups compared to surgery alone (*p* = 0.003). On multivariable analysis, chemotherapy was associated with significantly increased incidence of esophageal dilation (HR 2.9, 95% CI 1.5–5.5, *p* < 0.001) in oropharyngeal cancers.

**Conclusions:** The incidence of esophageal dilation is similar in LAHNC patients undergoing RT with or without surgery. Chemoradiotherapy increases the long-term risk of esophageal dilation events over surgery alone.

## Introduction

Head and neck cancer affects approximately 50,000 people each year in the US (1). In patients with locoregionally advanced head and neck cancer (LAHNC), it is often unclear whether to treat with primary chemoradiotherapy or primary surgical therapy with postoperative radiation or chemoradiation. Advantages to primary surgery include the additional prognostic information from surgical pathology and the ability to tailor adjuvant therapy according to surgical findings. However, an advantage of primary chemoradiotherapy is to obviate surgical morbidity in patients who are likely to require chemoradiotherapy anyway. Since patients with LAHNC, particularly those with human papilloma virus (HPV)-driven oropharyngeal primaries, frequently achieve good outcomes with either approach, long-term morbidity is a major factor in deliberating between treatment approaches, particularly in older patients.

Dysphagia is a principal chronic adverse effect of LAHNC treatment that can lead to feeding tube dependence, malnutrition, weight loss, aspiration pneumonia, and poor quality of life (2–5). The prevalence of significant swallowing dysfunction may be as high as 60%, and may lead to other adverse events, such as aspiration pneumonia requiring hospitalization (6–9). The etiology of chronic dysphagia is thought to be due to formation of radiation fibrosis, caused by chronic activation of myofibroblasts due to dysregulation of normal tissue repair mechanisms (10). Chemoradiotherapy, in particular, damages the pharyngeal constrictor muscles, leading to bolus stasis and risk for long-term aspiration (11). Consequently, upper esophageal strictures are reported in approximately 21% of patients undergoing chemoradiation (12).

Novel treatment approaches may limit dysphagia in patients with LAHNC, but how specifically radiation and surgery in combination affect stricture formation, and their relative contribution to the process, remain unclear. Minimally invasive surgical approaches, such as trans-oral robotic surgery (TORS) and trans-oral laser microsurgery (TLM) have been increasingly utilized, with uncertain effects on swallowing function (13, 14). Intensity-modulated radiation therapy (IMRT) allows for salivary gland-sparing, reducing xerostomia, which in turn correlates with dysphagia (15). Retrospective studies have also found that reducing dose to dysphagia related structures including the constrictor muscles, cricopharyngeal muscle, esophagus, and epiglottis can minimize swallowing dysfunction (16–19). Knowledge of the comparative effects on stricture formation could be helpful in comparing treatment alternatives and identifying effective strategies to reduce chronic dysphagia.

Prior population based studies (6) have used ICD-9 codes to identify dysphagia events, but this method is limited by variation in physician reporting and does not measure the severity of dysphagia. Moreover, given changes in radiation and surgical techniques over the past decade, there is a paucity of literature measuring long-term severe dysphagia incidence in the modern treatment era. The objective of this study was to compare severe swallowing dysfunction requiring esophageal dilation in the LAHNC population with various treatment approaches.

## Methods

### Population and sampling methods

This study was approved by the UC San Diego Institutional Review Board. We evaluated patients with LAHNC from the Surveillance, Epidemiology, and End Results (SEER)-Medicare linked database. The SEER program consists of a collection of cancer registries across the US which collect demographic, clinical, treatment, and survival information for individuals with cancer (20). The SEER-Medicare linkage combines longitudinal Medicare claims data for patients within the SEER database, providing a valuable resource to understand patterns of care and health outcomes for cancer patients from before diagnosis and throughout treatment, with complete follow-up extending through death.

We queried the SEER-Medicare database to identify patients over the age of 66 with LAHNC of the oral cavity, oropharynx, hypopharynx, larynx, or nasopharynx diagnosed between January 1, 2000 and December 31, 2009. Although Medicare coverage starts at 65, only patients over 66 are included to ensure a complete year of Medicare claims data to enable calculation of comorbidity scores. Subjects undergoing primary site surgery only, without either neck dissection or radiation, and those with non-squamous cell carcinoma histology, or multiple primary tumors, or diagnosis at autopsy or death were excluded. Subjects with missing date of diagnosis or with non-continuous part A and part B coverage or any part C enrollment (enrollment in an HMO) from 12 months prior to diagnosis to death or last follow-up were also excluded.

### Primary endpoint and covariate definitions

Esophageal dilation, the primary endpoint of this study, was identified using the following CPT codes: “43195,” “43196,” “43213,” “43214,” “43220,” “43221,” “43222,” “43223,” “43224,” “43225,” “43226,” “43233,” “43248,” “43249,” “43250,” “43451,” “43452,” “43453,” “43454,” “43455,” “43456,” “43457,” “43458,” “43459,” “43460,” “43510.” These represent different procedure codes for endoscopic esophageal dilation in the Medicare data. An esophageal dilation event was defined as the appearance of this CPT code in inpatient or outpatient Medicare billing claims. Time to an esophageal dilation event was defined from diagnosis date to the first esophageal dilation event, censoring at last follow-up or death.

The primary aim was to compare the cumulative incidence of esophageal dilation among LAHNC patients receiving definitive (chemo)radiation, vs. surgery and adjuvant (chemo)radiation, vs. surgery alone. Treatment information was obtained from both SEER and Medicare billing claims, using methods described previously (21–23).

Covariates included age at diagnosis (continuous), sex, race (black vs. non-black), marital status (married vs. unmarried), tumor site (oral cavity, nasopharynx, oropharynx, larynx, hypopharynx), stage (regional vs. local), and chemotherapy use (binary) (Table 1). The SEER Historic Stage A staging system was used to classify cancer stage, because TNM staging data is incomplete for the years studied and defined differently across sites.

**Table 1:** Sample descriptive statistics.

	**Total**	**RT**	**Surgery**	**Surgery with Adjuvant RT**	***P***
	**Mean (*****SD*****%)**	**Mean (*****SD*****%)**	**Mean (*****SD*****%)**	**Mean (*****SD*****%)**	
*N*	5,223	2,612 (50%)	*N* = 950 (18%)	*N* = 1,661 (32%)	
Age	75 (6.6)	76 (6.7)	76 (6.9)	75 (6.2)	
**TREATMENT**					< 0.0001
Chemotherapy	1,991 (62)	1,443 (55)	0 (0)	548 (33)	
No chemotherapy	3,232 (38)	1,169 (45)	950 (100)	1,113 (67)	
**DILATION STATUS**					< 0.0001
Dilation	361 (7)	211 (8)	30 (3)	120 (7)	
No dilation	4,862 (93)	2,401 (92)	920 (67)	1,541 (93)	
**SEX**					< 0.0001
Male	3,350 (64)	1,797 (69)	616 (54)	1,037 (62)	
Female	1,873 (36)	815 (31)	434 (46)	624 (38)	
**RACE**					0.003
White	4,518 (87)	2,243 (86)	834 (88)	1,441 (87)	
Black	397 (8)	229 (9)	56 (6)	112 (7)	
Asian	138 (3)	63 (2)	26 (3)	49 (3)	
Hispanic	81 (2)	43 (2)	8 (1)	30 (2)	
Other	89 (2)	34 (1)	26 (3)	29 (2)	
**MARITAL STATUS**					< 0.0001
Married	2,720 (52)	1,330 (51)	471 (50)	919 (55)	
Divorced	480 (9)	280 (11)	71 (7)	129 (8)	
Single	464 (9)	254 (10)	69 (7)	141 (8)	
Other	1,559 (30)	748 (29)	339 (36)	472 (28)	
**TUMOR SITE**					< 0.0001
Hypopharynx	479 (8)	343 (13)	23 (2)	104 (6)	
Larynx	1,100 (21)	624 (24)	81 (9)	395 (24)	
Nasopharynx	63 (1)	51 (2)	0 (0)	12 (1)	
Oral cavity	2,799 (54)	1,110 (43)	780 (82)	909 (55)	
Oropharynx	791 (15)	484 (66)	66 (7)	241 (15)	
**STAGE**					< 0.0001
Localized stage	1,592 (30)	654 (25)	421 (44)	517 (31)	
Regional stage	3,631 (70)	1,958 (75)	529 (56)	1,144 (69)	

### Statistical methods

We tested the null hypothesis that the unadjusted cumulative incidences of esophageal dilatation were equivalent within the three treatment groups, vs. the alternative hypothesis that the incidences were not equivalent, using Gray's test (24, 25) with Holm's *post-hoc* procedure for multiple comparisons (26). We also tested the null hypothesis that the cause-specific hazard ratio for esophageal dilation is equivalent in the three treatment groups (definitive (chemo)radiation, surgery and adjuvant (chemo)radiation, and surgery alone), using the Cox proportional hazards model, with surgery alone as the reference group, and controlling for the covariates mentioned above in the statistical model, with interaction terms included for chemotherapy and treatment group. All statistical tests were two-sided, with *p* < 0.05 indicating statistical significance. Analyses were conducted using SAS Enterprise Guide version 7.1.

## Results

**Figure 1:**
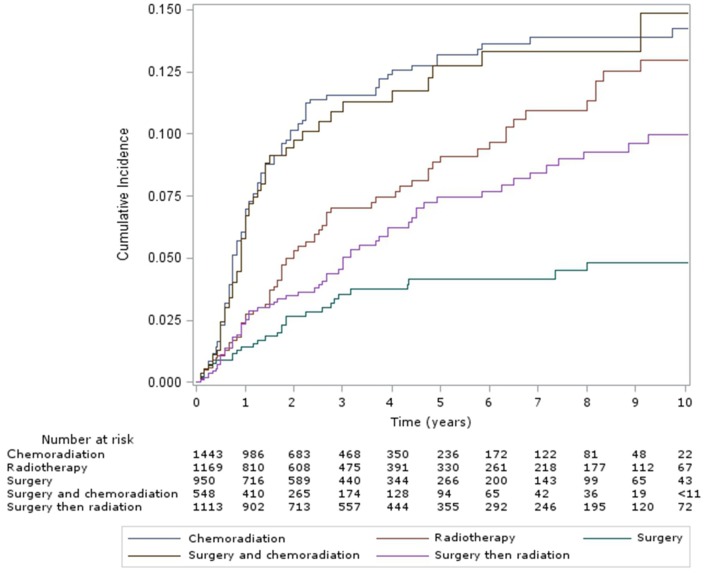
Cumulative incidence of esophageal dilation in all sites, by treatment group.

The cumulative incidence of esophageal dilation for all sites at 5 and 10 years, according to treatment group (Figure 1) were as follows – CRT: 13% (95% confidence interveal (CI) 11–15%) and 14% (95% CI, 12–17%), definitive RT: 9% (95% CI, 7–12%) and 13% (95% CI, 10–16%), surgery alone: 4% (95% CI, 3–6%) and 5% (95% CI, 3–7%), surgery and CRT: 13% (95% CI, 10–16%) and 15% (95% CI, 11–19%), and surgery and RT: 7% (95% CI, 6–10%) and 10% (95% CI, 8–13%). There was no significant difference in the incidence of esophageal dilation between surgery plus adjuvant RT/CRT or definitive RT/CRT (*p* = 0.37), but the incidence was significantly increased in both groups compared to surgery alone (*p* = 0.003). On multivariable analysis (Table 2), both definitive (HR 2.37, 95% CI 1.5–3.7, *p* < 0.001) and postoperative CRT (HR 2.85, 95% CI 1.8–4.6, *p* < 0.001) were associated with increased risk of esophageal dilation compared to surgery alone. In contrast, neither definitive nor postoperative RT was associated with significantly increased risk of dilation compared to surgery alone.

**Table 2:** Multivariable analysis of risk factors for esophageal dilation events.

	**Hazard ratio (95% CI)**	***P***
Age	1.00 (0.98, 1.02)	0.69
**SEX, REF: FEMALE**		
Male	1.09 (0.85, 1.40)	0.49
**RACE, REF: WHITE**		
Asian	0.88 (0.47, 1.63)	0.68
Black	0.98 (0.65, 1.47)	0.93
Hispanic	1.76 (0.94, 3.29)	0.08
Other	0.86 (0.35, 2.15)	0.75
**MARITAL STATUS, REF: MARRIED**		
Divorced	1.01 (0.69, 1.46)	0.98
Single	0.84 (0.56, 1.27)	0.41
Other	1.09 (0.84, 1.41)	0.53
**TUMOR SITE, REF: OROPHARYNX**		
Hypopharynx	2.84 (1.98, 4.09)	<**0.0001**
Larynx	1.84 (1.27, 2.67)	**0.0014**
Nasopharynx	0.74 (0.23, 2.39)	0.61
Oral cavity	0.97 (0.69, 1.35)	0.85
**TREATMENT, REF: SURGERY ALONE**		
Chemoradiation	2.37 (1.52, 3.70)	**0.0002**
Radiotherapy	1.46 (0.91, 2.34)	0.11
Surgery and Chemoradiation	2.85 (1.78, 4.57)	<**0.0001**
Surgery and radiotherapy	1.30 (0.83, 2.05)	0.26
**STAGE, REF: LOCALIZED**		
Regional	1.57 (1.19, 2.07)	**0.0016**

*Bold values are statistically significant (two-sided p < 0.05)*.

**Figure 2:**
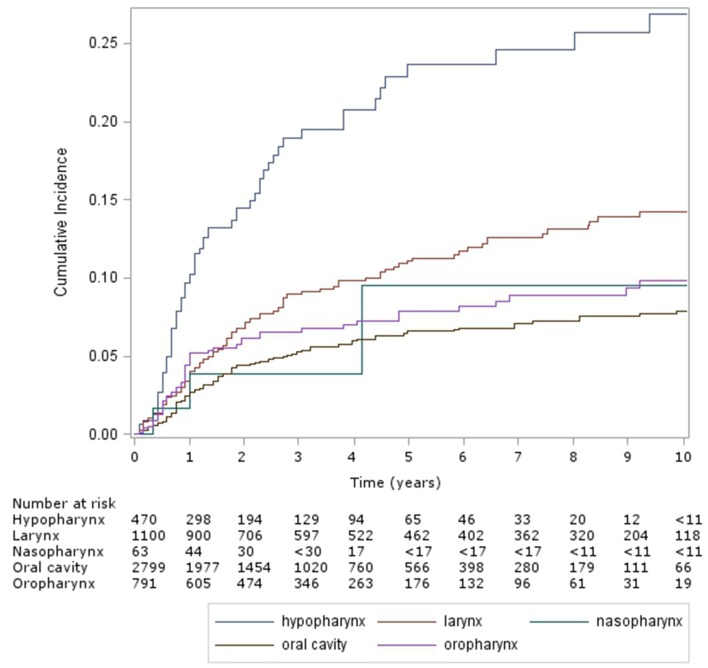
Cumulative incidence of esophageal dilation by site (Hypopharynx, Oral Cavity, Larynx, Nasopharynx, Oropharynx).

The cumulative incidence of esophageal dilation, according to site (Figure 2) were as follows – hypopharynx: 24% (95% CI 18–29%) and 27% (21–33%), larynx: 11% (9–14%) and 14% (12–17%), nasopharynx: 10% (2–26%) (and no events after 5 years), oral cavity: 7% (5–8%) and 8% (7–9%), and oropharynx: 8% (6–10%) and 10% (7–13%). On multivariable analysis (Table 2) factors associated with significantly increased risk of esophageal dilation were hypopharynx and larynx subsite and locoregionally advanced stage.

**Figure 3:**
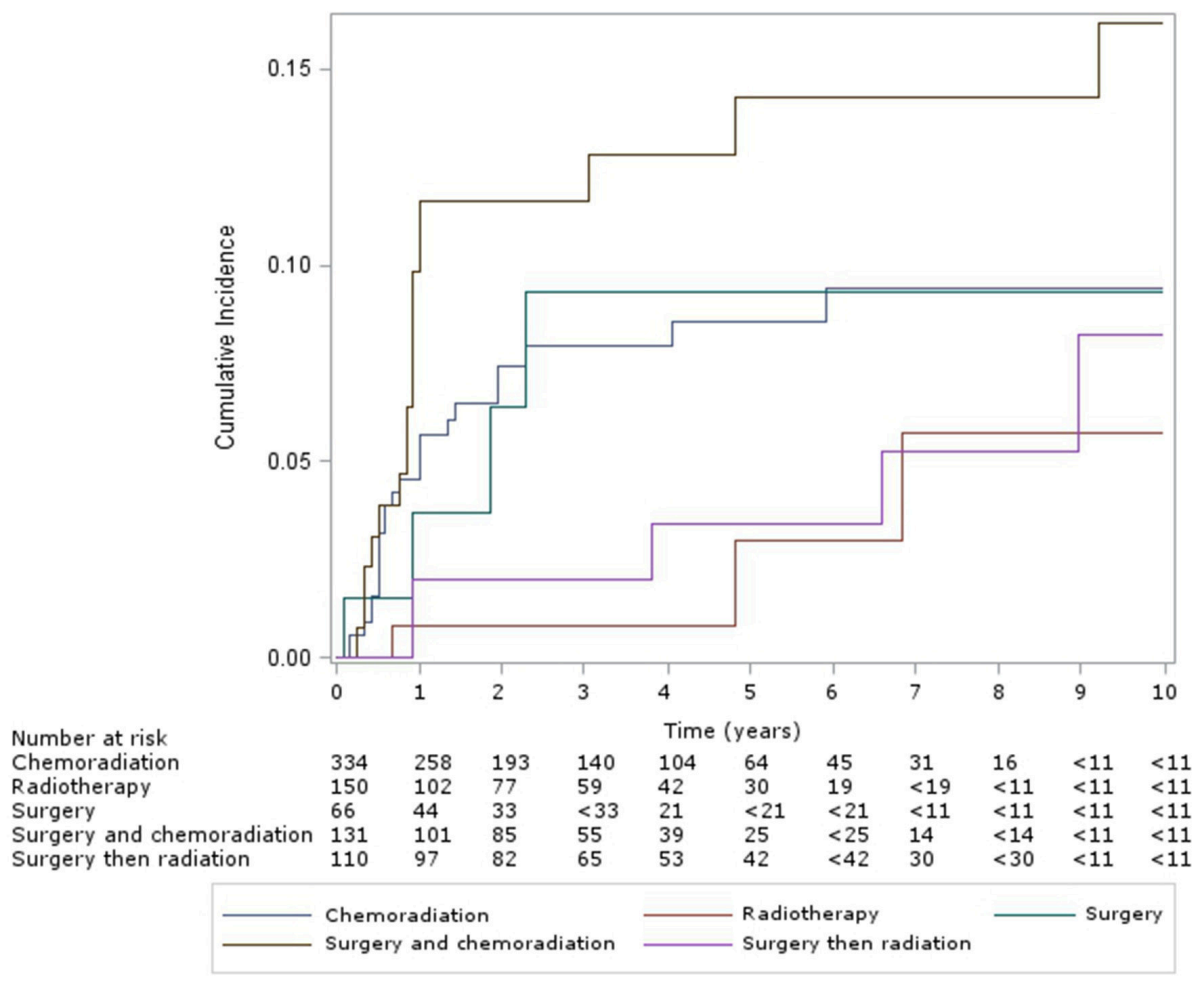
Cumulative incidence of esophageal dilation in oropharynx patients, by treatment group.

For patients with oropharyngeal subsite, the cumulative incidence of esophageal dilation for patients at 5 and 10 years, according to treatment group (Figure 3) were as follows – CRT: 9% (95% CI, 6–12%) and 10% (95% CI, 6–14%), definitive RT: 3% (95% CI, 0–10%) and 6% (95% CI, 1–2%), surgery alone: 9% (95% CI, 3–21%) – with no new events after 5 years, surgery and CRT: 14% (95% CI, 8–22%) and 16% (95% CI, 9–25%), and surgery and RT: 3% (95% CI, 1–9%) and 8% (95% CI, 3–18%). Multivariable analysis of this subgroup showed chemotherapy was associated with significantly increased incidence of esophageal dilation (HR 2.9, 95% CI 1.5–5.5, *p* < 0.001).

## Discussion

Management of head and neck cancer has rapidly changed over the past 20 years. Efforts to improve locoregional control and survival should be balanced with the need to maintain low long-term morbidity. Unfortunately, chemoradiation therapy is associated with significant detriment to quality of life, especially dysphagia (6–9). Efforts to minimize this are being investigated in prospective studies, including the ECOG E3311 and NRG HN-002 trials.

In our study we found that chemotherapy and radiation increased the hazard for esophageal dilation 4-fold compared to surgery alone, and that 15% of patients treated with surgery and adjuvant chemoradiation required esophageal dilation by 10 years. With surgery alone, the baseline incidence of dilations was approximately 5%, suggesting that the majority of the effect is attributable to radiation and chemotherapy. The finding that patients with locoregionally advanced hypopharynx, laryngeal and oropharyngeal disease had the greatest risk of dilation is consistent with this finding, since these tumors often are treated predominantly with chemoradiation. In contrast, most patients who receive surgery alone had localized oral cavity primaries, which reduces the risk for swallowing complications.

The mechanism by which chemotherapy would increase the risk of strictures is presumably due to radiosensitization of the normal tissues, though could also relate to its use in more advanced stages of disease, with increase in radiation dose volume to the esophagus. Given our findings, oncologists may want to consider the incremental benefit of combined modality approaches in elderly patients with advanced head and neck cancers. At a minimum, these data may be useful in counseling patients regarding their risk for esophageal strictures and other long-term sequelae of therapy, such as aspiration pneumonia. Targeting such patients for closer follow-up with swallowing studies and use of pneumococcal and influenza vaccination could be of value.

A strength of this study was our ability to analyze a relatively large population-based sample of patients at risk for high-grade esophageal complications, to examine risk factors for this event and to compare effects from alternative treatment approaches. Limitations include the fact that we were not able to examine effects on lower-grade events that may still have clinical significance. It is also possible that patients treated at high volume centers are more likely to be referred for esophageal dilation procedures, whereas patients treated at lower-volume centers may have limited access to such procedures, yet may still have severe dysphagia. In addition, the SEER-Medicare database lacks information regarding possible confounders, such as HPV status, radiation dose, type of surgery or chemotherapy dose or type information. It is likely that with increased IMRT utilization, swallowing structures are being preferentially spared, so radiation and chemoradiation may have less effect on chronic swallowing dysfunction in the future. Note that the conclusions of this study may have limited value for patients treated in the current era, particularly given the use of modern radiation techniques designed to avoid the pharyngeal constrictor muscles. Nevertheless, this study is helpful in quantifying the high probability of severe dysphagia requiring esophageal dilations in this population, and may aid future efforts aimed at reducing the risk of this complication in older patients.

## Conclusions

This study investigated the impact of different treatment approaches on the risk for esophageal dilation in the older LAHNC population. Our study documents the high probability of requiring esophageal dilation procedures after multi-modality treatment for LAHNC. In particular, radiosensitizing chemotherapy appears to significantly increase the probability of high-grade esophageal toxicity; thus caution should be given to the selection of elderly patients for chemoradiation. However, as more studies investigate the use of trans-oral robotic surgery, dose-reduced radiation schedules, and alternative systemic therapies, it is likely that significant reduction in toxicity and improvements in quality of life will be seen.

## Author contributions

All authors listed have made a substantial, direct and intellectual contribution to the work, and approved it for publication.

### Conflict of interest statement

The authors declare that the research was conducted in the absence of any commercial or financial relationships that could be construed as a potential conflict of interest.

## References

[1] Siegel R Naishadham D Jemal A. Cancer statistics, 2013. CA Cancer J Clin. (2013) 63:11–30. 10.3322/caac.21166 23335087

[2] Xu B Boero IJ Hwang L Le QT Moiseenko V Sanghvi PR. Aspiration pneumonia after concurrent chemoradiotherapy for head and neck cancer. Cancer (2014) 121:1303–11. 10.1002/cncr.29207 25537836 PMC4774546

[3] Daly ME Lau DH Farwell DG Luu Q Donald PJ Chen AM. Feasibility and toxicity of concurrent chemoradiation for elderly patients with head and neck cancer. Am J Otolaryngol. (2013) 34:631–5. 10.1016/j.amjoto.2013.07.010 23954137

[4] Caudell JJ Schaner PE Desmond RA Meredith RF Spencer SA Bonner JA. Dosimetric factors associated with long-term dysphagia after definitive radiotherapy for squamous cell carcinoma of the head and neck. Int J Radiat Oncol Biol Phys. (2010) 76:403–9. 10.1016/j.ijrobp.2009.02.017 19467801

[5] Gillespie MB Brodsky MB Day TA Lee FS Martin-Harris B. Swallowing-related quality of life after head and neck cancer treatment. Laryngoscope (2004) 114:1362–7. 10.1097/00005537-200408000-00008 15280708

[6] Francis DO Weymuller EA Jr Parvathaneni U Merati AL Yueh B Dysphagia, stricture, and pneumonia in head and neck cancer patients: does treatment modality matter? Ann Otol Rhinol Laryngol. (2010) 119:391–7. 10.1177/000348941011900605 20583737

[7] Smith RV Kotz T Beitler JJ Wadler S. Long-term swallowing problems after organ preservation therapy with concomitant radiation therapy and intravenous hydroxyurea. Arch Otolaryngol Head Neck Surg. (2000) 126:384–9. 10.1001/archotol.126.3.384 10722013

[8] Eisbruch A Lyden T Bradford CR Dawson LA Haxer MJ Miller AE. Objective assessment of swallowing dysfunction and aspiration after radiation concurrent with chemotherapy for head-and-neck cancer. Int J Radiat Oncol Biol Phys. (2002) 53:23–8. 10.1016/S0360-3016(02)02712-8 12007937

[9] Rose BS Jeong JH Nath SK Lu SM Mell LK. Population-based study of competing mortality in head and neck cancer. J Clin Oncol. (2011) 29:3503–9. 10.1200/JCO.2011.35.7301 21844503

[10] Martin M Lefaix J Delanian S. TGF-beta1 and radiation fibrosis: a master switch and a specific therapeutic target? Int J Radiat Oncol Biol Phys. (2000) 47:277–90. 10.1016/S0360-3016(00)00435-1 10802350

[11] Lazarus CL Logemann JA Pauloski BR Colangelo LA Kahrilas PJ Mittal BB. Swallowing disorders in head and neck cancer patients treated with radiotherapy and adjuvant chemotherapy. Laryngoscope (1996) 106:1157–66. 10.1097/00005537-199609000-00021 8822723

[12] Lee WT Akst LM Adelstein DJ Saxton JP Wood BG Strome M. Risk factors for hypopharyngeal/upper esophageal stricture formation after concurrent chemoradiation. Head Neck. (2006) 28:808–12. 10.1002/hed.20427 16732601

[13] Chung TK Rosenthal EL Magnuson JS Carroll WR. Transoral robotic surgery for oropharyngeal and tongue cancer in the United States. Laryngoscope (2015) 125:140-5. 10.1002/lary.24870 25093603 PMC4347815

[14] Hutcheson KA Holsinger FC Kupferman ME Lewin JS. Functional outcomes after TORS for oropharyngeal cancer: a systematic review. Eur Arch Otorhinolaryngol. (2015) 272:463–71. 10.1007/s00405-014-2985-7 24643851 PMC4169348

[15] Logemann JA Pauloski BR Rademaker AW Lazarus CL Mittal B Gaziano J. Xerostomia: 12-month changes in saliva production and its relationship to perception and performance of swallow function, oral intake, and diet after chemoradiation. Head Neck. (2003) 25:432–7. 10.1002/hed.10255 12784234

[16] Eisbruch A Schwartz M Rasch C Vineberg K Damen E Van As CJ Dysphagia and aspiration after chemoradiotherapy for head-and-neck cancer: which anatomic structures are affected and can they be spared by IMRT? Int J Radiat Oncol Biol Phys. (2004) 60:1425–39. 10.1016/j.ijrobp.2004.05.050 15590174

[17] Levendag PC Teguh DN Voet P van der Est H Noever I de Kruijf WJ. Dysphagia disorders in patients with cancer of the oropharynx are significantly affected by the radiation therapy dose to the superior and middle constrictor muscle: a dose-effect relationship. Radiother Oncol. (2007) 85:64–73. 10.1016/j.radonc.2007.07.009 17714815

[18] Caglar HB Tishler RB Othus M Burke E Li Y Goguen L. Dose to larynx predicts for swallowing complications after intensity-modulated radiotherapy. Int J Radiat Oncol Biol Phys. (2008) 72:1110–8. 10.1016/j.ijrobp.2008.02.048 18468812

[19] Feng FY Kim HM Lyden TH Haxer MJ Feng M Worden FP. Intensity-modulated radiotherapy of head and neck cancer aiming to reduce dysphagia: early dose-effect relationships for the swallowing structures. Int J Radiat Oncol Biol Phys. (2007) 68:1289–98. 10.1016/j.ijrobp.2007.02.049 17560051

[20] Warren JL Klabunde CN Schrag D Bach PB Riley GF. Overview of the SEER-Medicare data: content, research applications, and generalizability to the United States elderly population. Med Care (2002) 40:IV-3-18. 10.1097/01.MLR.0000020942.47004.03 12187163

[21] Warren JL Harlan LC Fahey A Virnig BA Freeman JL Klabunde CN. Utility of the SEER-Medicare data to identify chemotherapy use. Med Care (2002) 40:55–61. 10.1097/01.MLR.0000020944.17670.D7 12187169

[22] Virnig BA Warren JL Cooper GS Klabunde CN Schussler N Freeman J. Studying radiation therapy using SEER-Medicare-linked data. Med Care (2002) 40:49–54. 10.1097/00005650-200208001-00007 12187168

[23] Deyo RA Cherkin DC Ciol MA Adapting a clinical comorbidity index for use with ICD-9-CM administrative databases J Clin Epidemiol. (1992) 45:613–9. 10.1016/0895-4356(92)90133-8 1607900

[24] Fine JP Gray RJ A proportional hazards model for the subdistribution of a competing risk. J Am Stat Assoc. (1999) 94:496–509. 10.1080/01621459.1999.10474144

[25] Gray RJ A class of K-sample tests for comparing the cumulative incidence of a competing risk. Ann Stat. (1988) 16:1141–54. 10.1214/aos/1176350951

[26] Dunnett CW A multiple comparison procedure for comparing several treatments with a control. J Am Stat Assoc. (1955) 50:1096-121.

